# Research on the Micro Sheet Stamping Process Using Plasticine as Soft Punch

**DOI:** 10.3390/ma7064118

**Published:** 2014-05-27

**Authors:** Xiao Wang, Di Zhang, Chunxing Gu, Zongbao Shen, Huixia Liu

**Affiliations:** School of Mechanical Engineering, Jiangsu University, Xuefu Road, Zhenjiang 212013, Jiangsu, China; E-Mails: dizhang@outlook.com (D.Z.); chunxinggu@hotmail.com (C.G.); szb@ujs.edu.cn (Z.S.); lhx@ujs.edu.cn (H.L.)

**Keywords:** sheet forming, plasticine, soft punch, stamping, material flow

## Abstract

Plasticine is widely used in the analysis of metal forming processes, due to its excellent material flow ability. In this study, plasticine is used as the soft punch to fabricate array micro-channels on metal sheet in the micro sheet stamping process. This is because plasticine can produce a large material flow after being subjected to force and through the material flow, the plasticine can cause the sheet to fill into the micro-channels of the rigid die, leading to the generation of micro-channels in the sheet. The distribution of array micro-channels was investigated as well as the influence of load forces on the sheet deformations. It was found that the depth of micro-channels increases as the load force increases. When the load force reaches a certain level, a crack can be observed. The micro sheet stamping process was also investigated by the method of numerical simulation. The obtained experimental and numerical results for the stamping process showed that they were in good agreement. Additionally, from the simulation results, it can be seen that the corner region of the micro-channel-shape work piece has a risk to crack due to the existence of maximum von Mises stress and significant thinning.

## 1. Introduction

With the requirements of miniaturization in many industrial clusters, there is a growing demand for micro parts. Metallic micro-parts are becoming more and more important in practical applications due to their mechanical properties and thermal stability which are superior to those of polymeric micro-parts [1]. As a promising approach to manufacture micro-sheet parts, micro-sheet forming processes have been a research hot spot for manufacturers and researchers [2,3,4,5,6]. There are many advantages in the process: such as high productivity, low production cost, low energy consumption, good mechanical properties and stable dimensional accuracy.

However, in such processes it would be hard and complex to design and manufacture the rigid die and punch because accurate assembly between the rigid punch and die should be guaranteed, as when traditional rigid punch forming technology is used to deform the metal sheet. Therefore, micro sheet stamping processes based on the soft punch are employed. It is easy to assemble the punch and rigid die precisely after using soft material as the punch in the process [7]. The cost and time taken in the design and manufacture of the punch can be reduced in this way.

The sheet soft punch stamping process has been a subject of investigation. Browne and Battikha [8] presented an experimental study of the rubber forming process in order to produce sheet metal components. Sala [9] studied the influence of some signiﬁcant parameters on the flexible forming process by numerical simulation. Several kinds of flexible forming processes to produce sheet metal ashtrays and plates were presented by Thiruvarudchelvan [10]. Dirikolu *et al.* [11] optimized the rubber forming process based on numerical simulations and experiments. Peng’s research group [7] investigated a kind of micro sheet soft punch stamping process to fabricate micro channels via numerical simulations and experiments. According to the reported processes above, the early researchers tended to utilize hyper-elastic material as the punch due to its hyper-elastic and incompressible characteristics. Moreover, the use of flexible rubber material in direct contact with the work piece surface would endow the parts with good surface quality, because no tool marks would be created. However, the present application of elastic material as the soft punch in the sheet soft punch stamping process still has some problems: the spring back deformation of the work piece would be enhanced because the elastic material for the soft punch always tries to release the elastic deformation and retracts to its original position once the load is removed. Moreover, in the stamping process, the low level of material flow ability would make it hard to fabricate the micro-features in the sheet, such as the deep micro-channels, because the traditional elastic material is hard to get to flow into the micro-features of the rigid die.

In order to overcome these drawbacks, plastic material may well be a potential choice as the soft punch. Plasticine, the most widely used soft modeling material, which shows elastic-plastic flow behavior and excellent material flow ability, has been successfully used as a convenient model material to simulate plastic deformation of metals in metal forming processes for the last 40–50 years [12,13,14,15]. By undergoing large plastic deformation with the deforming metal sheets, plasticine can reduce the spring back deformation of metal sheets. Meanwhile, it can also be considered as a damage-less forming process because plasticine as soft punch is softer than metal sheets and other rigid molds. Moreover, as the soft punch, plasticine can producea large material flow after being subjected to force. Through the material flow, plasticine can make the sheet fill into the micro-features, resulting in the generation of micro-features in the sheet. Since plasticine has the required suitable material properties, a large interest in applications of plasticine in the sheet soft punch stamping process has been reported.

In this paper, plasticine is used as the soft punch to fabricate array micro-channels on metal sheet. The physics of the micro sheet stamping process using plasticine as soft punch is studied along with the influence of load forces on the deformations. The experimental results on the distribution of array micro-channels are also presented. The implicit procedure of ANSYS ﬁnite element code was employed to better understand the process as well as to evaluate the risk of cracks.

## 2. Experiment Setup

**Figure 1:**
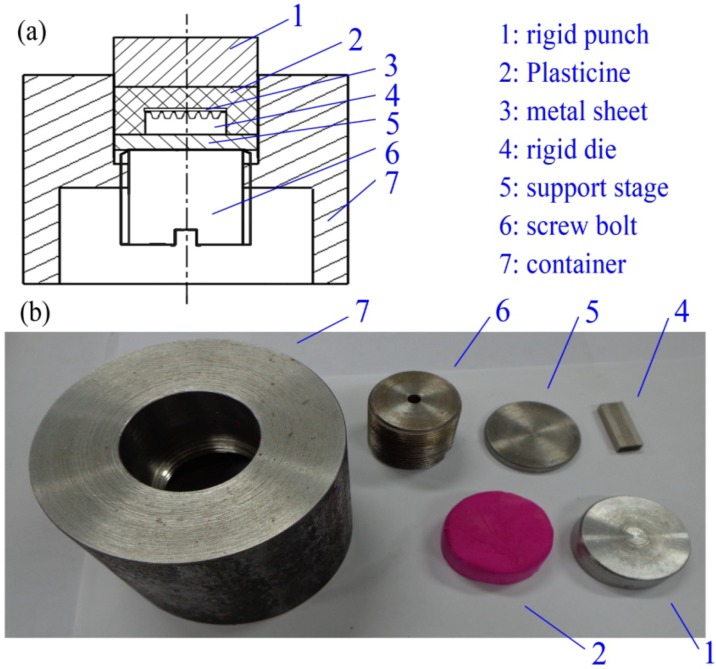
Experiment setup of the micro sheet stamping process using plasticine as soft punch.

**Figure 2:**
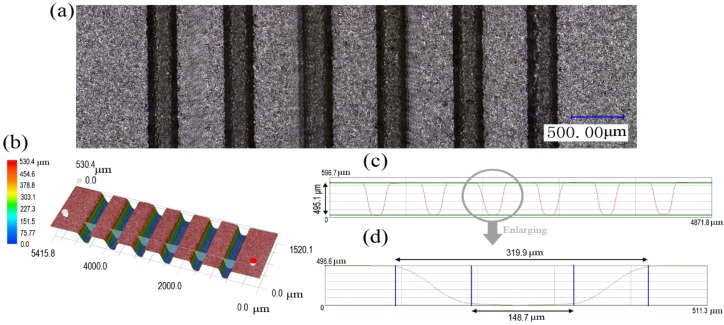
The shape of the rigid die: (**a**) the 2D plot of the rigid die; (**b**) the 3D plot of the rigid die; (**c**) the profile curve of the whole array micro-channels; (**d**) the profile curve of one micro-channel with detailed dimensions.

In order to study the characteristics of the micro sheet stamping process using plasticine as soft punch, the experimental setup was prepared as shown in Figure 1. The experimental assembly is made up of a rigid punch, plasticine, metal sheet, a rigid die, a support stage, a screw bolt and a container. The rigid die was manufactured by electrical-discharge wire cutting (wire EDM). As shown in Figure 2, six array channels with the same dimensions were fabricated on the rigid die. The surface of the rigid die has a good surface quality.

In this research, pure aluminum (10 μm in thickness), which has an excellent formability and wide applications in micro parts fabrication, was selected as the work piece. The red colored plasticine (made by CYMO) was chosen as the soft punch to transfer the forming force. Since commercially available plasticine contains a considerable amount of air pockets, these air pockets should be squeezed out of the material in order to establish a reasonable degree of homogeneity. After removing the air pockets by working the material through repeated rolling and folding operations, the plasticine material was cut into pieces, 40 mm in diameter and 10 mm in height by using a plexiglass “cookie cutter”. The cylindrical-shaped plasticine was placed in the rigid place, as shown in Figure 1. Additionally, to detach the plasticine from the surfaces of the metal sheet after stamping, the PE-membrane (8 μm) was placed between the plasticine and work piece in the experiment.

**Figure 3:**
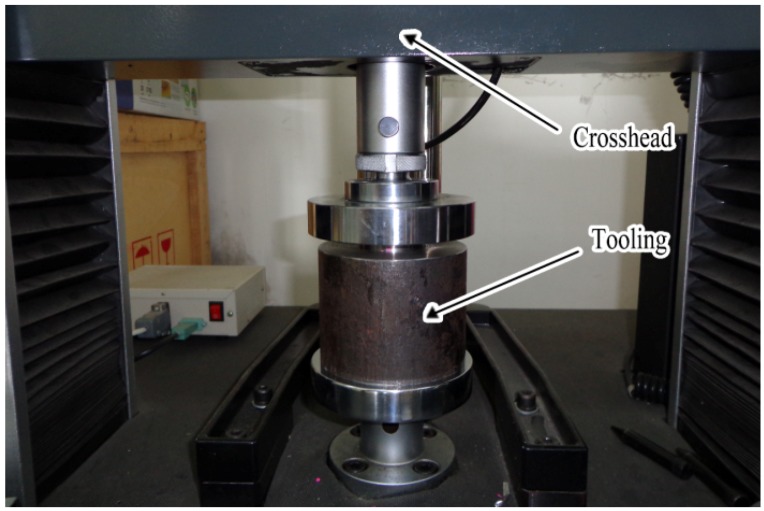
Micro sheet stamping platform.

Figure 3 shows the testing platform. The tooling was assembled by the rigid punch, plasticine, metal sheet, rigid die, support stage, screw bolt and container and was then placed on the testing platform. A slow compression speed of 5 mm/min was used in this research. The data of the displacements and forces could be obtained from the sensors on the testing platform.

## 3. Experimental Results and Discussions

**Figure 4:**
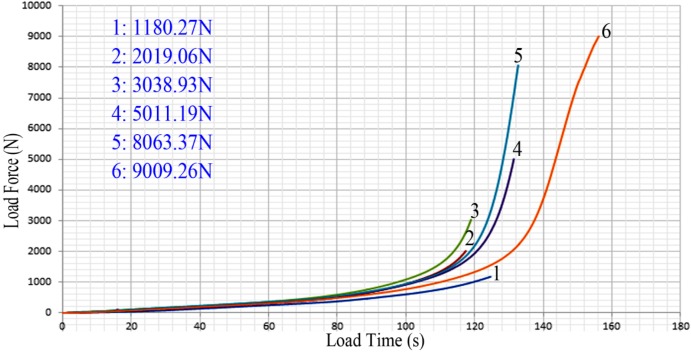
The load curves.

In order to investigate the influence of load forces on deformation behaviors, the maximum load forces were set as 1000 N, 2000 N, 3000 N, 5000 N, 8000 N and 9000 N. That is to say, in our experiments, the loading was stopped once the load force reached the set values described above. Figure 4 shows the different load curves when the maximum force data were 1180.27 N, 2019.06 N, 3038.93 N, 5011.19 N, 8063.37 N and 9009.26 N, respectively. From Figure 4, it can be observed that the different load curves with various maximum load forces do not coincide with each other. However, the trend of the different load curves is the same. According to Figure 4, there is a gradual increase followed by a more rapid increase in the value of the force. With increasing time, the rigid punch becomes loaded down and the gap between the rigid die and plasticine is reduced. Finally, plasticine fills the gap and becomes a medium to transfer the forming force, leading to the generation of an array micro-channels on the work piece. In addition, the different increase rates of the curves can be explained by the fact that different size gaps exist due to the placement of the plasticine.

**Figure 5:**
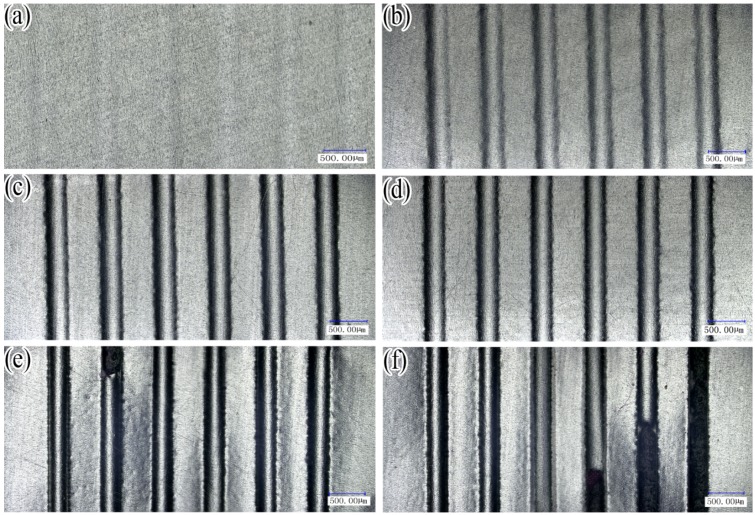
2D topography of the metal sheet surfaces: (**a**) with the final load force 1180.27 N; (**b**) with the final load force 2019.06 N; (**c**) with the final load force 3038.06 N; (**d**) 5011.10 N; (**e**) 8063.37 N; (**f**) 9009.26 N.

Figure 5 shows the 2D topography of the metal sheet surfaces with the various final load forces. The experimental results were observed with a digital microscope (Keyence VHX-1000). As shown in Figure 5a, a shallow channel was obtained, but it is hard to see. From Figure 5b–d, the formed six micro-channels become easier to see, meaning that the depths of the channels are increased with the force of the load. According to the results, the surfaces of micro-channels have a good surface quality, revealing that the stamping process can be considered as non-damage forming because the plasticine as soft punch is softer than the metal sheets and the other rigid dies. With increasing load, the sheet metal generates cracks as shown in Figure 5e,f. It can be implied that the metal sheet reaches the limit of forming with increasing load. Besides, the distribution of cracks should be noted. The material properties of aluminum sheet used in the experiment may be responsible for the generation and distribution of cracks.

**Figure 6:**
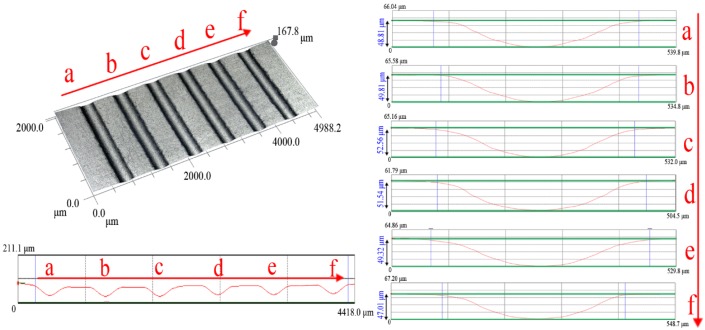
The 3D topography of the formed channels with the final load force 5011.19 N, the profile curves of the whole array micro-channel features (six channels, marked with a,b,c,d,e,f) with final load force 5011.19 N and the enlarged graphs of six profile curves of micro-channels (marked with a,b,c,d,e,f) with the final load force 5011.19 N.

Since six array micro-channels were formed with the help of the rigid die after stamping, before going into a critical discussion of the influence of load forces on deformation behaviors, it is necessary to understand the data on the array micro-channel distribution. Figure 6 indicates the profile curves distribution of each channel, meaning that the whole six array channels can be obtained in this way. Seen from the measurement data, there are a few changes in the depth values. The channels located in the middle region of the sheet have larger depth values than those located at the edge region of sheet. One reason for this phenomenon can be explained as follows: since plasticine is placed between the rigid punch and metal sheet and filled into the container, the existence of friction between the container wall and the plasticine may be responsible for the uneven distribution of the array micro-channels depths. When the punch moves down, the center zone of the plasticine is free to deform and can only be affected by the surrounding material. However, in the surrounding zone, the friction on the container wall resists the material flow. It leads to the different material flow rates existing in the center and edge zones of the plasticine. Finally, the aluminum sheet then forms different depths of micro-channels.

**Figure 7:**
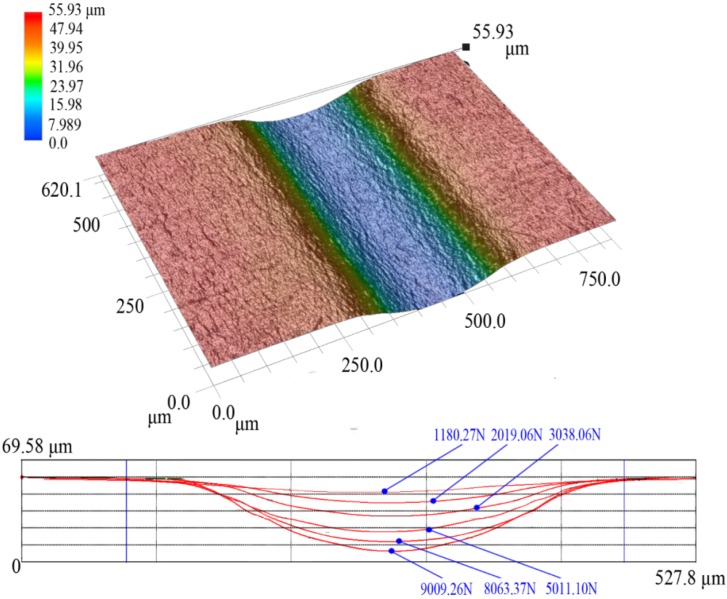
One magniﬁcation micro-topography of the micro-channel located in the middle region of the metal sheet and six profile curves of channels with different final load forces.

It is important to highlight the influence of load forces on the profile curves of the formed channels. Figure 7 describes one 3D plot of a micro-channel (channel c) located in the middle region of the metal sheet. It also shows the profile curves of channels with different final load forces. According to the results, the widths of the micro-channels remain unchanged and the depths are increased. It is obvious that the depths of micro-channels would be increased along with increase in the final load forces.

## 4. Numerical Simulation

In an attempt to simulate the micro sheet stamping process with different final load forces, the implicit procedure of ANSYS ﬁnite element code was employed.

### 4.1. Modeling Requirements

There are two different material models in the FEA model. One is the elastic-plastic model of metal sheets and the other is the elastic-linear strain hardening material model to describe the deformation of plasticine. In order to obtain accurate results, precise material models should be inputted into the numerical model.

#### 4.1.1. Metal Sheet Material Constitutive Model

As mentioned above, 10 μm thick commercially supplied aluminum sheets were used in our experiments. The micro sheet stamping process is a quasi-static process with low value and small change of strain-rates, some assumptions are applied in the modelling such as: materials can be modelled as elastic/perfectly plastic, all plastic deformation occurs at the same low strain-rate, and a linear equation of state is valid [16]. Therefore, to describe the aluminum sheets’ deformation, the elastic/perfectly plastic material model was chosen and employed in the numerical simulation. This model does not incorporate any strain hardening parameters. Additionally, the von Mises strength model is used. It can be deﬁned using the materials shear modulus and yield strength.

#### 4.1.2. Elastic-Linear Strain Hardening Material Model for Plasticine

Plasticine is a proprietary material, which has nonlinear stress-strain characteristics for relatively large deformations. The common use of plasticine does not demand a strict control of properties. As a consequence, the behavior of plasticine may vary from batch-to-batch, often in an unpredictable manner. The constitutive model selection has been a subject of investigation for many researchers in the past [14]. There are two main constitutive models to describe ﬂow behavior of plasticine. One is described by use of an elastic-plastic model while the other one is elasto-viscoplastic model. In several studies, the ﬂow behavior of plasticine was assumed to be elastic-plastic represented by the well-known power law (Hollomen) equation given by σ = Kε^*n*^ where K is the strength coefficient and n is the strain hardening exponent [13,15,17]. In other papers, a ﬁnite element simulation of squeeze flow or upsetting has been carried out for a paste material, namely plasticine, for which material behavior was described by an elasto-viscoplastic constitutive model [18,19]. In our ﬁnite element analysis, the elastic-plastic behavior assumed for material and true stress-true strain relation, was presented in the form of the well-known power law (Hollomen) equation.

**Figure 8:**
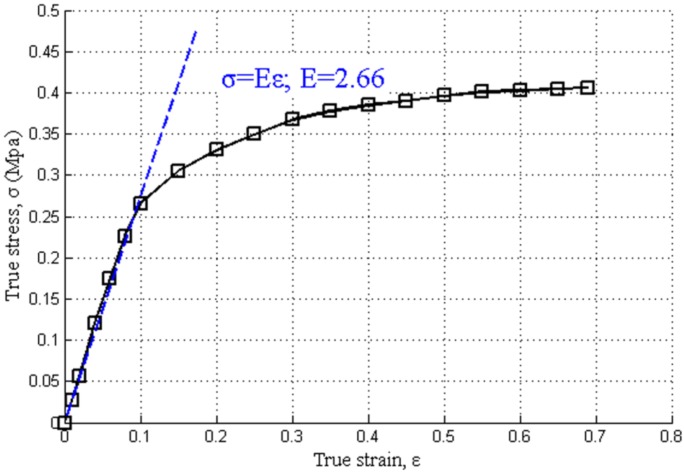
The true stress-true strain curve of red plasticine in our experiment.

The compression tests were carried out using red plasticine (Cylinder: 12 mm in diameter and 30 mm in height) at the same constant crosshead speed of 5 mm/min in order to determine its mechanical properties. In these uniaxial compression experiments, data were taken in the form of compression load against the deformation of plasticine. Later on, these data were used to obtain the true stress-true strain curve of plasticine. Figure 8 shows the true stress-true strain curve of red plasticine. The obtained curves were then directly inputted into the program of the FEA as the true stress-true strain data.

### 4.2. Finite Element Model

**Figure 9:**
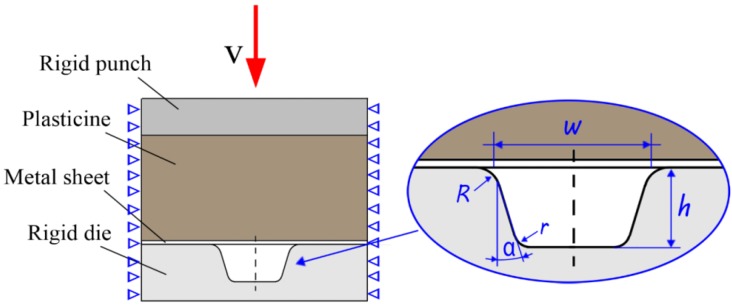
The sketch of the shape and geometric dimensions of the model.

In this paper, numerical simulations and experiments were done to demonstrate the feasibility of the micro sheet stamping process using plasticine as the soft punch. It can be figured out that every micro-channel is independent under symmetrical loading during the stamping process. Therefore, one micro-channel was extracted to establish the FEA model to simulate the micro sheet stamping process. Figure 9 shows the sketch of the shape and geometric dimensions of the model. The numerical model from top to bottom is as follows: rigid punch, plasticine, thin metal sheet and rigid die. The values of the key geometric dimensions are as follows:

Micro draw angle: α = 10°;
Upper radius of the rigid die: *R* = 40 μm;
Lower radius of the rigid die: *r* = 20 μm;
Width of the micro groove: *w* = 320 μm;
Depth of the micro groove: *h* = 500 μm.

The elastic-linear strain hardening material is deﬁned for plasticine with size 0.8 mm × 0.72 mm (The number of total elements in the model is 1020), and the elastic/ perfectly plastic model is deﬁned for 10 μm thickness aluminum sheets with size 0.8 mm × 0.01 mm (The number of total elements in the model is 640). The plasticine and the thin aluminum sheet were modeled by utilizing PLANE 182 element which is a two dimensional four-node element. The rigid punch and die are deﬁned with several lines and arc elements of type TARGE 169. The contacts between the surface of the plasticine and metal sheet and the surface of the rigid punch and die were modeled with the three-point element elements of CONTA 172. Symmetrical boundary conditions were deﬁned according to the actual bound conditions. Additionally, the rigid punch should be loaded with a constant speed of 5.0 mm/min and to meet the actual situation of the experiments, the friction coefficient was set to 0.18 since Vaseline was used in the stamping experiments as a lubricant.

### 4.3. Simulation Results and Discussions

**Figure 10:**
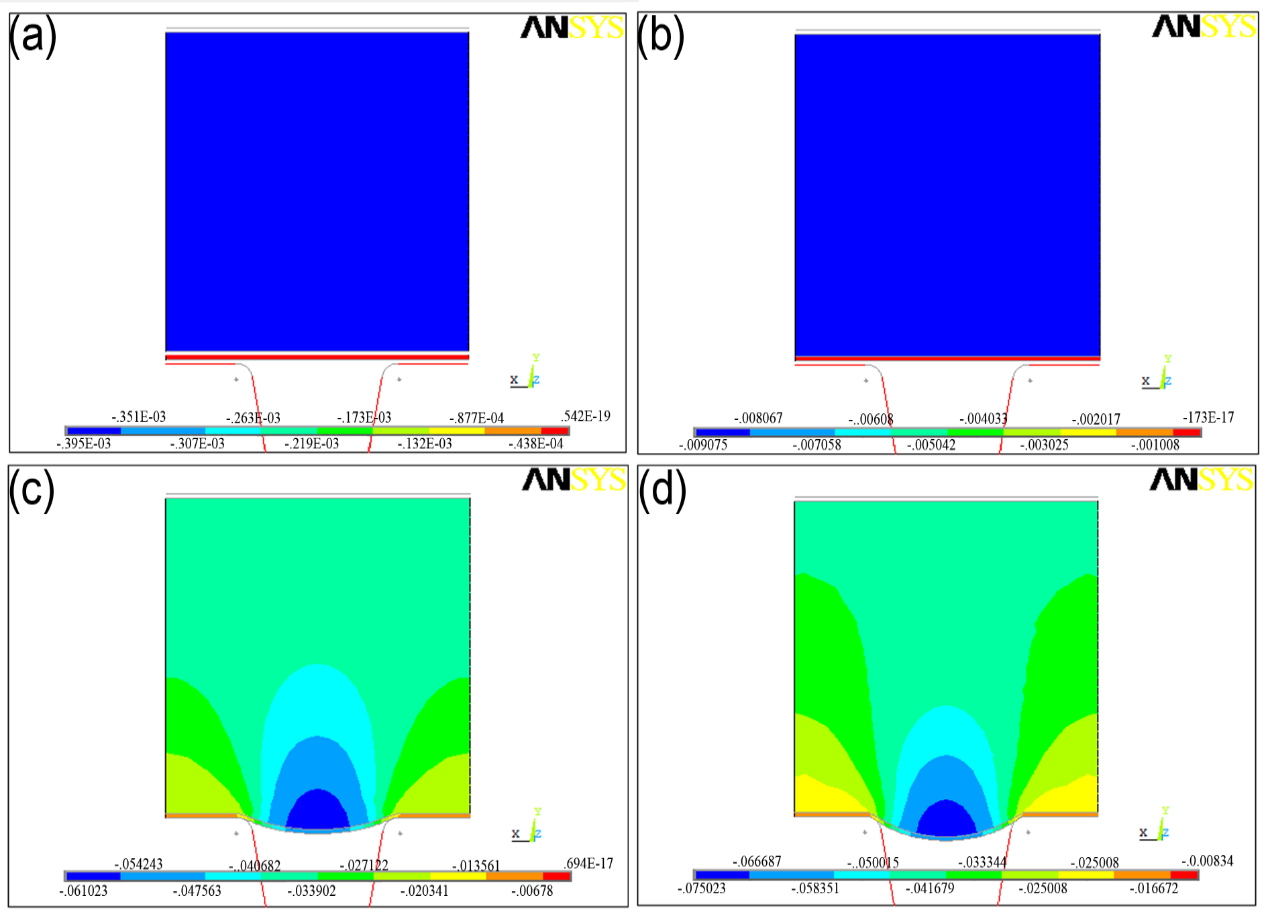
The typical stages of deformations with contours of Y-displacement distribution (mm).

Figure 10 shows the micro sheet stamping process step by step. Figure 10a show the state at the initial moment and Figure 10d displays the final state. From the simulation results, the micro sheet stamping process may be divided into two different stages: the ﬁrst is the plasticine self-deformation stage, as shown in Figure 10b; the second is the metal sheets’ deformation under the pressure of plasticine to move downward together with the ﬁlling of the metal sheets until they reach the form limit, as shown in Figure 10c,d. During stamping, rigid punch movement, plasticine deformation, plasticine stamping with the metal sheet, and bending processes occur in that sequence. As shown in Figure 10d, it can be seen that the maximum deformation occurred at the center of the sheet.

**Figure 11:**
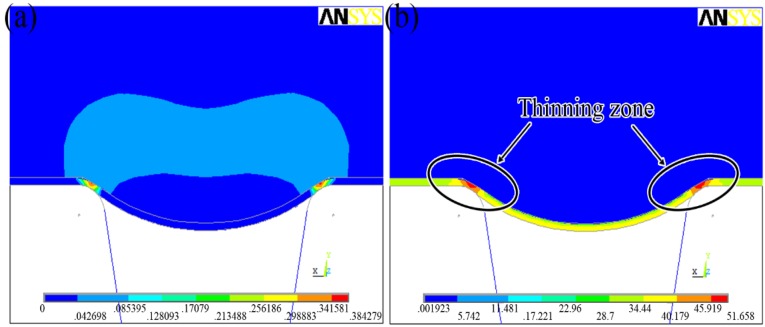
(**a**) The final deformation with the contour of effective plastic strain distribution; (**b**) The final deformation with contour of von Mises stress distribution.

Figure 11a shows the final deformation with the contour of effective plastic strain distribution. From the results, it can be found that ﬁnite element analysis can predict the ﬁnal shape of the work piece properly and the corner region of channel-shape work piece has the maximum effective plastic strain. Figure 11b indicates the final deformation with the contour of von Mises stress distribution. The maximum von Mises stress was also observed in the corner region of the channel-shape work piece. Since the von Mises stress distribution can be used to evaluate the risk of sheet cracks, it can be inferred that the cracks observed in the experiments would occur in the maximum von Mises stress zone. Moreover, when the punch moves down, the plasticine makes the metal sheet to fill into the micro-channels of the rigid die, the outside material of the sheet is bent, and then drawn into the die cavity. The inner material is pushed downwards and the tension force is applied at the corner region. The interfacial friction resists the material ﬂow and further accelerates the material thinning. As shown in Figure 11b, the thin zone of sheet is located in the corner region, which is regarded as easy to generate the crack and have the risk of sheet cracks.

**Figure 12:**
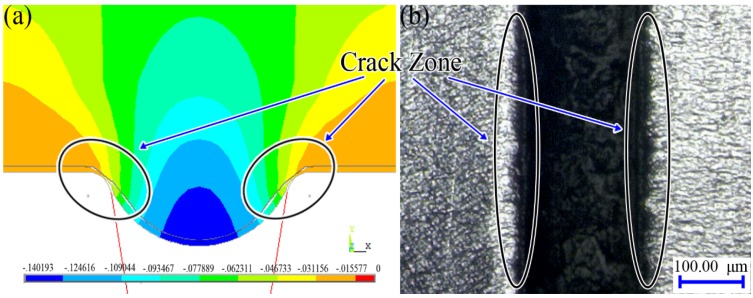
(**a**) The mode with the contour of Y-displacement distribution (mm) after using the method of the birth-death element; (**b**) the photograph of the crack generated in the experiment.

In order to find out the zone of crack, the birth-death element method was used. The elements which have stress values that exceed the failure stress would be removed in the simulation. Figure 12a shows the mode with the contour of Y-displacement distribution after using the method of the birth-death element. It is obvious that the material of the work piece near the corner of the rigid die becomes thin. The corner region of the channel-shape work piece is the dangerous area where a crack is generated. As shown in Figure 12b, the crack zone generated in the experiment (Location: channel f; Load force: 9009.26 N) is the same as the simulation results, which shows the effectiveness of the simulation.

**Figure 13:**
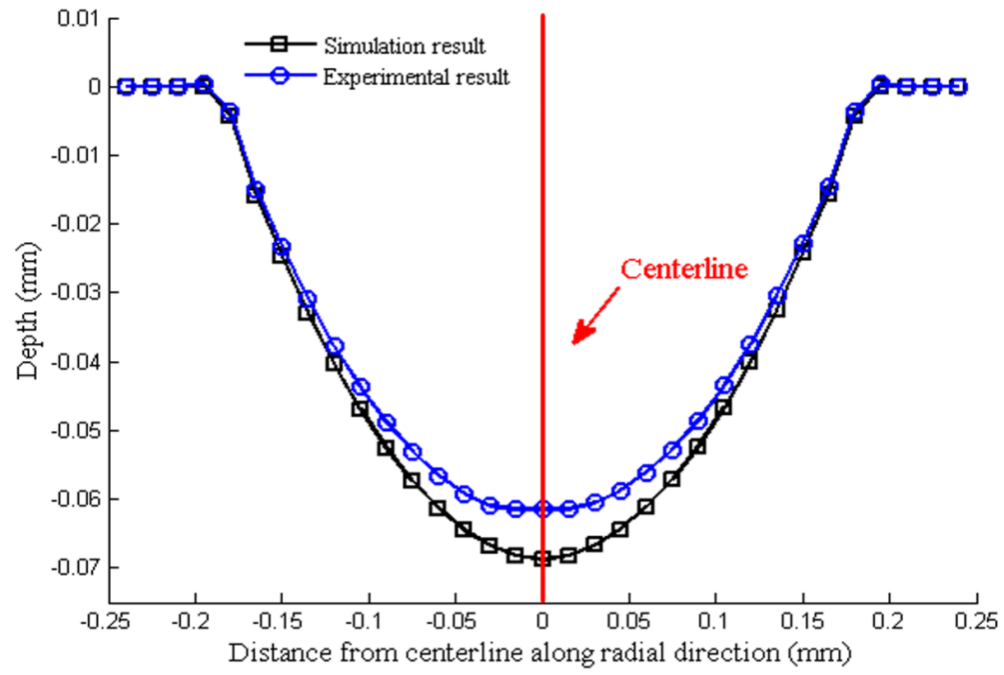
Comparison of the micro-crater proﬁles between the experimental and the numerical results.

Figure 13 shows the comparison of the micro-channel depths between the experimental and the numerical results. According to the results, the maximum deformation depth of the micro-channel obtained by simulation can reach as deep as 68.83 μm while the depth obtained by experiment reaches only 61.48 μm even when the final load value is set as 9000 N. As shown in Figure 5f, half of the six formed channels in the work piece have generated cracks, meaning that the metal sheets have reached the forming limit with the final load force 9009.26 N. Although the experimental data are limited due to the restriction on the adjustment of final load forces, the existing results of micro-channel depths imply that ﬁnite element analysis can properly predict the ﬁnal shapes of micro-channels in the work piece. It is obvious that the simulation results are in good agreement with the experiment, yet some errors still exist. The simplified symmetric model and the used material properties of aluminum may be responsible for the relatively larger simulation results.

## 5. Conclusions

In this paper, the micro sheet stamping process using plasticine as soft punch was studied with experiments and simulations along with the influence of load forces on the sheet deformations. The following conclusions can be made according to the experimental and simulated results:

(1) By experiment, the whole six array micro-channels can be obtained. It was observed that the surfaces of the micro-channels possess good surface quality. Since the plasticine for the soft punch is softer than the metal sheets and other rigid dies, the stamping process can be considered as a non-damage forming process.
(2) The depths of the channels are increased with increase in the final load forces. When the final load force reaches a certain level, cracks can be observed. Additionally, according to the distribution of the array micro-channels as well as the depth changes, it can be assumed that the existence of friction between the container wall and the plasticine is responsible for the uneven distribution of the array micro-channel depths.
(3) According to the simulation results, the micro sheet stamping process may be divided into two different stages: the ﬁrst is the plasticine self-deformation stage; the second is the metal sheets’ deformation under the pressure of plasticine to move downward with ﬁlling of the metal sheets until they reach the form limit.
(4) Based on the contour of von Mises stress distribution, the maximum von Mises stress of the work piece occurred in the corner region of the work piece. During the process, the outside material of the sheet is bent, and then drawn into the die cavity. The inner material is pushed downwards and the tension force is applied at the corner region. The interfacial friction resists the material ﬂow and further accelerates the material thinning. The thin zone of the sheet is located in the corner region which is the same as the maximum von Mises stress zone. It is easy to generate a crack and have the risk of sheet cracks.
